# Community surveillance of COVID-19 by village health volunteers, Thailand

**DOI:** 10.2471/BLT.20.274308

**Published:** 2021-03-02

**Authors:** Nayawadee Kaweenuttayanon, Ratrawee Pattanarattanamolee, Nithikorn Sorncha, Shinji Nakahara

**Affiliations:** a Khon Kaen Regional Hospital, Khon Kaen, Thailand.; b Graduate School of Health Innovation, Kanagawa University of Human Services, 3-25-10 Tonomchi, Kawasaki, Kanagawa 210-0821, Japan.

## Abstract

**Problem:**

To control the increasing spread of coronavirus disease 2019 (COVID-19), the government of Thailand enforced the closure of public and business areas in Bangkok on 22 March 2020. As a result, large numbers of unemployed workers returned to their hometowns during April 2020, increasing the risk of spreading the virus across the entire country.

**Approach:**

In anticipation of the large-scale movement of unemployed workers, the Thai government trained existing village health volunteers to recognize the symptoms of COVID-19 and educate members of their communities. Provincial health offices assembled COVID-19 surveillance teams of these volunteers to identify returnees from high-risk areas, encourage self-quarantine for 14 days, and monitor and report the development of any relevant symptoms.

**Local setting:**

Despite a significant and recent expansion of the health-care workforce to meet sustainable development goal targets, there still exists a shortage of professional health personnel in rural areas of Thailand. To compensate for this, the primary health-care system includes trained village health volunteers who provide basic health care to their communities.

**Relevant changes:**

Village health volunteers visited more than 14 million households during March and April 2020. Volunteers identified and monitored 809 911 returnees, and referred a total of 3346 symptomatic patients to hospitals by 13 July 2020.

**Lessons learnt:**

The timely mobilization of Thailand’s trusted village health volunteers, educated and experienced in infectious disease surveillance, enabled the robust response of the country to the COVID-19 pandemic. The virus was initially contained without the use of a costly country-wide lockdown or widespread testing.

## Résumé

**Problème:**

Pour lutter contre la propagation croissante de la maladie à coronavirus (COVID-19), le gouvernement de Thaïlande a ordonné la fermeture des espaces publics et commerciaux à Bangkok le 22 mars 2020. Par conséquent, de nombreux travailleurs au chômage sont retournés dans leurs contrées natales au cours du mois d'avril 2020, augmentant le risque de dissémination du virus dans l'ensemble du pays.

**Approche:**

En prévision de ces vastes mouvements de travailleurs au chômage, le gouvernement thaïlandais a formé des volontaires de la santé déjà présents dans les villages afin qu'ils puissent détecter les symptômes de la COVID-19 et sensibiliser les membres de leur communauté. Les bureaux provinciaux de la santé ont constitué des équipes de surveillance de la COVID-19 composées de ces volontaires en vue d'identifier les personnes revenant des zones à haut risque, d'encourager le respect d'une quarantaine spontanée de 14 jours, mais aussi de contrôler et signaler l'apparition de tout symptôme suspect.

**Environnement local:**

Malgré un renforcement récent du contingent de soignants dans le but de répondre aux objectifs de développement durable, la pénurie de professionnels de santé subsiste dans les régions rurales de la Thaïlande. Pour y remédier, le système de soins de santé primaires fait appel à des volontaires spécialement formés dans les villages, qui prodiguent des soins de base aux membres de leur communauté.

**Changements significatifs:**

Les volontaires de santé dans les villages ont rendu visite à plus de 14 millions de foyers en mars et avril 2020. Ils ont identifié et suivi 809 911 rapatriés et orienté un total de 3346 patients symptomatiques vers les hôpitaux au 13 juillet 2020.

**Leçons tirées:**

La mobilisation rapide de volontaires de santé fiables dans les villages thaïlandais, formés et habitués à surveiller les maladies infectieuses, a permis au pays de réagir avec fermeté à la pandémie de COVID-19. Dans un premier temps, la Thaïlande a pu maîtriser le virus sans imposer un confinement coûteux sur tout le territoire ni recourir à une campagne de dépistage à grande échelle.

## Resumen

**Situación:**

Para controlar la creciente propagación de la enfermedad por coronavirus (COVID-19), el gobierno de Tailandia impuso el cierre de las zonas públicas y comerciales de Bangkok el 22 de marzo de 2020. Como resultado, un gran número de trabajadores desempleados regresaron a sus ciudades de origen durante abril de 2020, lo que aumentó el riesgo de propagación del virus en todo el país.

**Enfoque:**

En previsión del movimiento a gran escala de trabajadores desempleados, el gobierno tailandés formó a los voluntarios sanitarios de las aldeas existentes para que reconocieran los síntomas de la COVID-19 y educaran a los miembros de sus comunidades. Las oficinas provinciales de salud formaron equipos de control de la COVID-19 con estos voluntarios para identificar a los que regresaban de las zonas de alto riesgo, fomentar la autocuarentena durante 14 días y controlar e informar sobre la aparición de cualquier síntoma relevante.

**Marco regional:**

A pesar de la importante y reciente ampliación del personal sanitario para alcanzar las metas de los objetivos de desarrollo sostenible, sigue habiendo escasez de personal sanitario profesional en las zonas rurales de Tailandia. Para compensar esta carencia, el sistema de atención primaria de salud incluye voluntarios sanitarios formados en las aldeas que prestan atención sanitaria básica a sus comunidades.

**Cambios importantes:**

Los voluntarios sanitarios de las aldeas visitaron más de 14 millones de hogares durante marzo y abril de 2020. Los voluntarios identificaron y controlaron a 809.911 repatriados, y remitieron a los hospitales a un total de 3.346 pacientes sintomáticos hasta el 13 de julio de 2020.

**Lecciones aprendidas:**

La oportuna movilización de los voluntarios sanitarios de confianza de las aldeas tailandesas, formados y con experiencia en el control de enfermedades infecciosas, permitió la sólida respuesta del país a la pandemia de la COVID-19. En un principio, el virus se contuvo sin necesidad de recurrir a un costoso bloqueo en todo el país ni a pruebas generalizadas

## ملخص

المشكلة للتحكم في التفشي المتزايد للمرض الناتج عن فيروس كورونا (كوفيد 19) فرضت حكومة تايلند إغلاق الأماكن العامة وأماكن الأعمال التجارية في بانكوك يوم 22 مارس/آذار 2020. كانت نتيجة ذلك أن عادت أعداد كبيرة من العاطلين عن العمل إلى بلداتهم الأصلية خلال شهر أبريل/نيسان 2020، مما رفع خطر انتشار الفيروس على مستوى البلد بأكملها.

الأسلوب بناء على توقع حكومة تايلند للانتقال واسع النطاق للعاطلين عن العمل، أجرت الحكومة تدريبًا للمتطوعين الصحيين القرويين الموجودين للتعرف على أعراض كوفيد 19 وتوعية أفراد مجتمعاتهم. أنشأت مكاتب الصحة الإقليمية فرقًا رقابية لمرض كوفيد 19 من هؤلاء المتطوعين لتتعرف على العائدين من المناطق عالية الخطر وتشجع على العزل الذاتي لمدة 14 يومًا وتراقب ظهور أي أعراض ذات صلة وتبلغ بالتطورات.

المواقع المحلية على الرغم من التوسع الكبير مؤخرًا في القوى العاملة في مجال الرعاية الصحية لتحقيق أهداف التنمية المستدامة، ما زال هناك نقص في الأخصائيين الصحيين المحترفين في المناطق الريفية في تايلند. لتعويض هذا النقص، يتضمن نظام الرعاية الصحية الأساسية متطوعين صحيين قرويين مدربين يقدمون الرعاية الصحية الأساسية لمجتمعاتهم.

التغيّرات ذات الصلة قام المتطوعون الصحيون القرويون بزيارات لأكثر من 14 مليون منزل خلال شهري مارس/آذار وأبريل/نيسان 2020. حدد المتطوعون 809911 من العائدين وراقبوهم وأحالوا إجمالاً 3346 مريضًا تظهر عليهم الأعراض إلى المستشفيات حتى يوم 13 يوليو/تموز 2020.

الدروس المستفادة التعبئة في الوقت المناسب للمتطوعين الصحيين القرويين محل الثقة في تايلند ـ والذين لديهم الوعي والخبرة في مجال الرقابة على الأمراض المعدية ـ أدت إلى استجابة قوية من البلد لجائحة كوفيد 19. تم احتواء الفيروس مبدئيًا دون اللجوء إلى إغلاق مكلف على مستوى البلد أو اختبارات واسعة النطاق.

## 摘要

**问题:**

为控制冠状病毒病（新型冠状病毒肺炎）的不断蔓延，泰国政府于 2020 年 3 月 22 日强制关闭了曼谷的公共场所和商业区域。因此，在 2020 年 4 月期间，大量失业工人返乡导致该病毒在全国范围内传播的风险加剧。

**方法:**

考虑到失业工人的大规模流动，泰国政府对现有的乡村卫生志愿者进行了培训，以确保其能够识别新型冠状病毒肺炎的症状，并对其社区成员进行教育。各省卫生厅组织这些志愿者组成新型冠状病毒肺炎监测小组，对来自疫情高发地区的返乡人员进行识别，鼓励其自我隔离 14 天，并对相关症状的发展进行监测和报告。

**当地状况:**

尽管为了实现可持续发展目标，医疗人员队伍已于最近进行了大幅度扩大，但是泰国农村地区仍然缺乏专业医疗人员。为了弥补这一点，初级卫生保健系统将训练有素的乡村卫生志愿者纳入其中，以安排其为社区提供基本的卫生保健服务。

**相关变化:**

2020 年 3 月至 4 月，乡村卫生志愿者走访了 1400 多万户家庭。截至 2020 年 7 月 13 日，志愿者确定并监测了 809 911 名返乡人员，并已将总计 3346 名有症状的患者转诊到医院。

**经验教训:**

泰国的乡村卫生志愿者在传染病监测方面受过培训且经验丰富，应及时动员这些值得信赖的志愿者，以确保该国能够有效应对新型冠状病毒肺炎疫情。最初并没有使用代价高昂的全国封锁或大规模检测来控制病毒。

## Резюме

**Проблема:**

С целью контроля над растущим распространением коронавирусной болезни (COVID-19) правительство Таиланда приказало закрыть общественные места и деловые кварталы в Бангкоке с 22 марта 2020 года. В результате множество безработных вернулись домой в апреле 2020 года, увеличив тем самым риск распространения вируса по всей стране.

**Подход:**

Предвидя масштабное перемещение безработных, тайское правительство обучило имеющихся в сельской местности добровольцев системы здравоохранения тому, как распознавать симптомы COVID-19 и распространять нужные знания среди местных жителей. Органы здравоохранения на уровне провинций собрали из этих добровольцев группы надзора за распространением инфекции COVID-19, задачей которых было выявлять лиц, вернувшихся из зон высокого риска, поощрять среди них самоизоляцию на 14 дней, а также отслеживать развитие любых релевантных симптомов и сообщать об этом.

**Местные условия:**

Несмотря на то что в последнее время количество медицинских работников в стране значительно возросло в связи с необходимостью достижения целей в области устойчивого развития в сельских районах Таиланда все еще отмечается нехватка профессиональных медработников. В целях компенсации нехватки кадров система первичной медико-санитарной помощи включает в себя также деятельность обученных деревенских добровольцев, оказывающих базовую медицинскую помощь жителям своих общин.

**Осуществленные перемены:**

Деревенские добровольцы системы здравоохранения посетили более 14 миллионов семей в течение марта и апреля 2020 г. Они выявили и отслеживали 809 911 лиц, вернувшихся домой, и направили в общей сложности 3346 лиц с симптомами в больницу по состоянию на 13 июля 2020 г.

**Выводы:**

Своевременная мобилизация в Таиланде надежных добровольцев системы здравоохранения, обученных надзору за проявлениями инфекционного заболевания и опытных в этом вопросе, позволила стране уверенно противостоять пандемии COVID-19. На начальном этапе вирус удалось локализовать без дорогостоящих мер, таких как локдаун в масштабе всей страны или всеобщее тестирование.

## Introduction

**Fig. 1:**
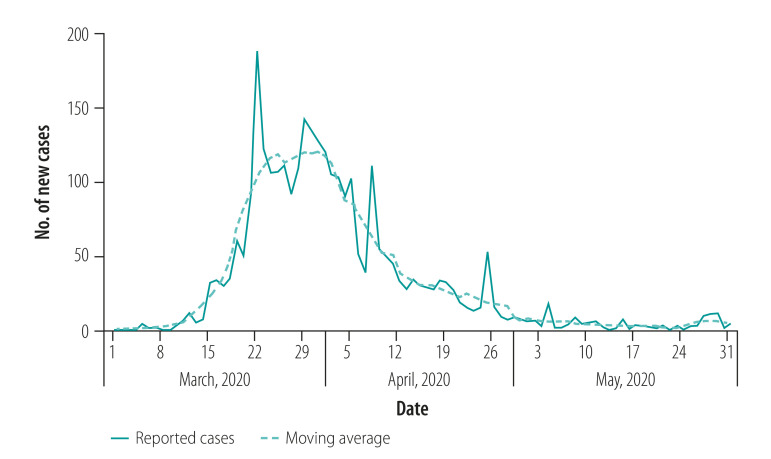
Daily number of new cases of coronavirus disease 2019, Thailand, March–May 2020

Source: Department of Disease Control, Ministry of Public Health, Thailand.^1^

Thailand reported the first case of coronavirus disease 2019 (COVID-19) on 13 January 2020. On 3 March 2020, the Thai government initiated a mandatory 14-day quarantine for all travellers from countries with high numbers of new cases, and began to promote preventive measures such as the wearing of face masks and regular washing of hands. The number of new cases peaked at 188 per day on 22 March as a result of three clusters of super-spreaders in Bangkok and the southern provinces (Fig. 1). The Thai government contained the transmission and the success was attributable to the primary health-care system, including community (village) health volunteers.^2^^–^^4^ We describe this unique aspect of the health-care system in Thailand and its effect on the response to the pandemic. We demonstrate how the exhaustive surveillance conducted by the volunteers, an approach that goes much further than tracing the contacts of COVID-19-positive cases, was effective in containing the virus.^5^

## Local setting

### Primary health care 

To provide basic health care to rural areas lacking professional health-care personnel, the Thai health authorities use village health volunteers from local communities. Village leaders and existing volunteers recruit volunteers, who receive an intensive week of training at local health centres in health education, health promotion, disease prevention and basic medical treatment. Since 2004, when volunteers contributed to avian influenza surveillance, training in infectious disease surveillance has also been included.^4^ The training is funded by the public health ministry, the sub-district administrative office and provincial health offices, and delivered by local health officers or district or provincial hospital staff. Volunteers are subsequently supervised by sub-district-level health officials. In the situation where volunteers encounter health problems beyond their care abilities, they refer individuals to a health centre or a hospital via health officials.

Over 1 million village health volunteers currently serve their communities, with each volunteer covering five to 15 households within their neighbourhood. The amount of time donated by volunteers varies from a few hours per week to long shifts over a period of several days, depending on the health situation and the particular province. Through their work, the volunteers obtain an awareness of the health-care needs of their community and develop a close relationship with their beneficiaries.^4^ Volunteers receive 1000 Thai Bhat (equivalent to 32 United States dollars or around 3 days’ minimum wage) per month from the government towards expenses (e.g. travel costs), and this was temporarily increased by 50% during March–December 2020. Other incentives include: discounted hospital costs for volunteers and their families; the opportunity for the offspring of volunteers to become health-care students; the potential honour of receiving a Royal Decoration; or simply the opportunity to serve their communities.^6^

### COVID-19 response

The Thai government closed all schools on 18 March 2020 and all Bangkok public and business areas on 22 March 2020, declared a state of emergency and enforced a curfew over all of Thailand on 26 March 2020, and suspended the arrivals of all international commercial flights on 4 April 2020. To avoid a large increase in unemployment in the capital as a result of these restrictions, the government allowed interprovincial travel during April. Thai citizens working abroad were also permitted to return to Thailand and, after a mandatory 14-day quarantine in Bangkok, to their hometowns. However, the nationwide movement of people in large numbers (at the peak of interprovincial travel, 70 000–80 000 unemployed returned to their hometowns per day) increased the risk of spreading the virus across the entire country. Workers attempting to return to their provinces were therefore screened at airports and train and bus stations;^7^ any showing symptoms were referred to the nearest hospital for further testing. 

## Approach

In preparation for the large-scale movement of people across the country, the public health ministry delivered online courses and local health officers provided in-person training for the volunteers.^2^^,^^8^ The training included basic knowledge of COVID-19, educating the population in how to stay safe, identifying and monitoring members of the community at high risk (either older people or those with chronic illness such as cardiovascular disease, diabetes or hypertension), and data collection and reporting. Although volunteers could submit hard copies of records, the public health ministry strongly encouraged the online reporting of data using their specially designed smartphone or internet application (app). 

After the effective closure of Bangkok, the National Communicable Disease Committee instructed provincial governors to mobilize community health resources to limit the spread of COVID-19 across the country.^7^ The provincial health offices assembled local COVID-19 surveillance teams of village health volunteers in all sub-districts. The volunteers were provided with personal protective equipment, such as face shields or goggles, head covers, surgical masks, gloves and raincoats (as substitutes for gowns), by the central and local governments, health offices and in donations from their community. 

The local surveillance teams (i) educated their community members about the disease, preventive measures and relevant symptoms to self-monitor and report; (ii) identified and monitored the returnees from Bangkok or abroad, as well as members of the community classified as being at high risk (i.e. recorded temperature and any other symptoms); (iii) reported the list of those being monitored to the sub-district health officials; and (iv) notified the sub-district health officials of symptomatic patients for further action (i.e. referral to a designated hospital for testing and initiating of contact-tracing procedures if positive).

Instead of relying on the contact-tracing approach that is only used when a positive case is newly identified, volunteer surveillance teams adopted a strategy involving exhaustive monitoring of individuals arriving from Bangkok and abroad.^9^ Volunteers visited their allocated households and requested that any returnees, if encountered, self-quarantine for 14 days at home. The surveillance teams noted the medical histories of any returnees, and encouraged them to report any symptoms daily using a smartphone app.^2^ If returnees were not able to use the app, the volunteers conducted daily household visits to record any symptoms while wearing personal protective equipment. Volunteers also continued their usual routine of monitoring the health conditions of other people within their allocated households.

Volunteers used a web-based COVID-19 surveillance database, developed by the public health ministry and password-protected to maintain confidentiality, to register all the returnees, returnees who developed symptoms, close contacts of confirmed cases and groups considered to be at high risk.^2^^,^^10^ In the case where volunteers preferred to complete physical printed forms, local health officers uploaded data to the online system on behalf of the volunteers. The database was also used to record volunteer activities such as household visits for monitoring home quarantine, screening of groups at high risk at the designated areas in their community (e.g. village entrance, market or bus station), and supporting chronically ill or disabled patients. This record was also used as a performance report for each volunteer; maintaining volunteer status is conditional on achieving a satisfactory performance report.^2^

## Relevant changes

Village health volunteers visited more than 14 million households during the period of interprovincial travel (March to April 2020). The volunteers identified and monitored 809 911 returnees and 64 552 people at high risk, and referred a total of 3346 symptomatic patients to hospitals by 13 July 2020.^10^ The country-wide number of new cases steadily declined from the peak on 22 March 2020 to reach less than 10 new cases per day by 27 April 2020 (Fig. 1).

## Lessons learnt

In combination with an approach of exhaustive monitoring, the timely mobilization of Thailand’s village health volunteers was successful in containing the COVID-19 pandemic. The low number of COVID-19-positive cases in Thailand provides an indication of the robustness of the Thai health-care system in responding to public health emergencies. Volunteers have played a pivotal role in detecting and reporting unusual symptoms among animals and humans since the avian influenza outbreak in 2004;^4^ having these functional systems already in place enabled immediate activation of the required response and surveillance activities when the pandemic struck (Box 1).

Box 1 **Summary of key lessons learnt**

- The timely mobilization of Thailand’s village health volunteers, educated and experienced in infectious disease surveillance, enabled the robust response of the country’s health service to the COVID-19 pandemic.
- Following the return of those employed in Bangkok to their rural homes during the widespread closure of businesses, the exhaustive surveillance of all returnees by village health volunteers was crucial in containing the spread of the virus without a costly country-wide lockdown or widespread testing.
- The close relationship between the volunteer workforce and members of their rural communities enabled the smooth functioning of the disease surveillance, which might otherwise have been considered an intrusion of privacy.

COVID-19: coronavirus disease 2019.

Encouraged and advised by health volunteers, the prompt and thorough self-quarantine of returnees prevented the case numbers from surging as a result of interprovincial travel. Although this approach may be considered excessive by some members of the community, the observed containment of the virus could not have been achieved through case-based methods that require testing of symptomatic patients and epidemiological surveillance skills for contact-tracing.^9^ Although digital contact-tracing systems using mobile phones are available in Thailand, some people choose not to use these technologies or may not report positive test results because of their desire for privacy.^5^^,^^11^

Two important issues remain to be addressed. First, daily visits to potentially infected people may have caused stigmatization of the volunteers in some cases; this could have been addressed by enhanced personal protective equipment and raising awareness of the transmission mechanisms of the virus.^12^ Second, the important contribution of the volunteers, their labour-intensive role and their risk of exposure to infected individuals should be recognized. Although steps have been taken to address this issue – for example, the volunteers’ monthly allowance was temporarily increased during the height of the pandemic, hospital costs were discounted further for volunteers, and a small number of outstanding volunteers (< 20 per year) receive a Royal Decoration – whether these steps are sufficient is a topic under discussion.

The strategies adopted in Thailand were very different from those of other countries that achieved early containment of the outbreak; as opposed to strict travel restrictions or extensive COVID-19 testing,^13^^–^^15^ the exhaustive surveillance conducted by the village health volunteers was the most significant contribution to successful containment of the virus. The close relationship of the high-volume volunteer workforce with community members enabled the smooth functioning of the disease surveillance, which might otherwise have been considered an intrusion of privacy. We encourage other resource-constrained settings to consider such a response in future situations.
